# Study of CO_2_ Adsorption on Chemically Modified Activated Carbon With Nitric Acid and Ammonium Aqueous

**DOI:** 10.3389/fchem.2020.543452

**Published:** 2020-11-04

**Authors:** Liliana Giraldo, Diana Paola Vargas, Juan Carlos Moreno-Piraján

**Affiliations:** ^1^Departamento de Química, Facultad de Ciencias, Universidad Nacional de Colombia, Bogotá, Colombia; ^2^Departamento de Química, Facultad de Ciencias, Universidad del Tolima, Ibagué, Colombia; ^3^Departamento de Química, Facultad de Ciencias, Universidad de los Andes, Bogotá, Colombia

**Keywords:** activated carbon, chemical modification, nitric acid, ammonium aqueous, CO_2_ adsorption

## Abstract

The study of CO_2_ adsorption on adsorbent materials is a current topic of research interest. Although in real operating circumstances, the removal conditions of this gas is carried out at temperatures between 290 and 303 K and 1 Bar of pressure or high pressures, it is useful, as a preliminary approach, to determine CO_2_ adsorption capacity at 273K and 1 Bar and perform a thermodynamic study of the CO_2_ adsorption heats on carbonaceous materials prepared by chemical activation from African palm shell with CaCl_2_ and H_3_PO_4_ solutions, later modified with HNO_3_ and NH_4_OH, with the aim to establish the influence that these treatments have on the textural and chemical properties of the activated carbons and their relationship with the CO_2_ adsorption capacity. The carbonaceous materials were characterized by physical adsorption of N_2_ at 77K, CO_2_ at 273K, proximate analysis, Boehm titrations and immersion calorimetry in water and benzene. Activated carbons had a BET area between 634 and 865 m^2^g^−1^, with a micropore volume between 0.25 and 0.34 cm^3^g^−1^. The experimental results indicated that the modification of activated carbon with HNO_3_ and NH_4_OH generated a decrease in the surface area and pore volume of the material, as well as an increase in surface groups that favored the adsorption of CO_2_, which was evidenced by an increase in the adsorption capacity and the heat of adsorption.

## Introduction

The quality of the air that is breathed in almost the entire planet is being significantly affected by atmospheric pollutants present in daily anthropic activities, to which the supply and use of fossil fuels contributes approximately to 80% of the emissions of Greenhouse gases such as: carbon dioxide (CO_2_), methane (CH_4_) and nitrous oxide (N_2_O). From all the gases that affect the environment, CO_2_ could be the one that shows the greatest threat to the planet due to its ability to keep solar radiation inside the atmosphere causing an increase in global temperature.

This gas comes from natural sources such as: forest fires, volcanic eruptions, fossilization processes and animal respiration, as well as anthropogenic sources such as: burning of waste, burning of fossil fuels for obtaining energy, among other human activities (Koytsoumpa et al., 2018; Hussin and Aroua, 2019; Mardani et al., 2019). In recent years, carbon dioxide emissions into the atmosphere have increased dangerously, in this sense the global average concentrations of CO_2_ reached 405.5 (ppm) in 2017, compared to 403.3 ppm in 2016 and 400.1 ppm in 2015 according to the World Meteorological Organization (WMO), this increase is directly related to the increase in the temperature of the planet which is a problem of global concern that threatens the sustainability of life on earth (Rubino et al., 2019; World Meteorological Organization (WMO), 2019). Different international organizations have determined, that in order to reduce the impact that this situation has on living beings, ecosystems and sustainable development, it is necessary that the global temperature rise keeps below 1.5°C, therefore, mechanisms that allow the reduction of carbon dioxide and other greenhouse gas emissions in all sectors of society are needed (Rubino et al., 2019; World Meteorological Organization (WMO), 2019). According to above, technological options for CO_2_ capture have been studied, such as: chemical absorption, cryogenic separation, use of separation membranes, carbonation-calcination cycles and adsorption (Yu et al., 2012; Wang et al., 2017; Borhani and Wang, 2019). All of these have advantages and disadvantages associated with side effects in their implementation, operational design and costs. However, adsorption has been positioned as a process of interest for the capture of greenhouse gases, due to its characteristics and versatility. Specifically, it has been shown that studies aimed at the preparation and use of activated carbon for CO_2_ removal have increased in recent years, because this adsorbent material has a developed porous structure, a varied surface chemistry, a wide surface area, specificity, among other features that can be adjusted according to the needs of the application. For this, the preparation conditions of activated carbons can be selected, such as: the activating agents and their concentrations, the carbonization temperatures among other parameters. Additionally, the porous solids obtained can be subjected to chemical modifications to increase their affinity for an adsorbate of interest (Saha and Kienbaum, 2019). As mentioned above, this research work was carried out with the aim of determining the CO_2_ adsorption capacity at 273K and 1 Bar and performing a thermodynamic study of the CO_2_ adsorption heats on carbonaceous materials that were chemically modified, and this solid was obtained by using African palm shell, which is an agricultural waste of large production in Colombia. The precursor was chemically activated using two chemical agents mixed at different concentrations: CaCl_2_ and H_3_PO_4_, subsequently carbonized and the porous material obtained was chemically modified with HNO_3_ and NH_4_OH in order to enrich the surface chemistry of the solid with functional groups that increase its interaction with the CO_2_ molecule. The materials were texturally and chemically characterized using the following techniques: physical adsorption of N_2_ at 77K, CO_2_ at 273K, proximate analysis, Boehm titrations and immersion calorimetry in water and benzene. Finally, CO_2_ adsorption calorimetry was performed to determine energy aspects of the process.

## Materials and Methods

### Granular Activated Carbon Preparation

Granular activated carbon was prepared using as lignocellulosic precursor of African palm shell, an agricultural waste that is generated in Colombia as part of the productive chain of oil extraction. This material was washed, dried and led to a particle size between 3 and 4 mm; then, 50 g of the material were impregnated in 100 mL of a solution of CaCl_2_ (2%) and H_3_PO_4_ (32%) at a temperature of 358 K for 6 h (Nakagawa et al., 2007; Juárez-Galán et al., 2009), subsequently, the furnace temperature was increased to 393 K to dry the sample during an interval of 5 h. Subsequently, the carbonization process was carried out in a horizontal Carbolite furnace, at a temperature of 3 K min^−1^ with a CO_2_ flow of 100 mLmin^−1^ until reaching 1,073 K for 6 h, then it was changed to N_2_ flow and the temperature was decreased to 873 K, remaining constant for 2 h. Finally, the material obtained in the procedure described was labeled as GAC and it was subjected to a washing process with a solution of 0.01M HCl and hot distilled water until neutral pH.

### GAC Chemical Modification

The activated carbon called GAC, was divided into 2 equal parts and each part was subjected to different treatments.

#### Modification With Nitric Acid

Activated carbon (GAC) was put into contact with 100 mL of 6M HNO_3_ solution for 6 h at boiling temperature (Noh and Schwarz, 1990; Daud and Houshamnd, 2010). It was then filtered, washed with distilled water and finally dried at 373 K for 6 h. The resulting material was named as: GACO.

#### Modification With Ammonium Aqueous

Activated carbon (GAC) was put into contact with 100 mL of concentrated NH_4_OH at 353 K under reflux for 24 h (Plaza et al., 2011). It was then filtered, washed with distilled water and finally dried at 373 K for 6 h. The resulting material was named as: GACA.

### Characterization of Carbonaceous Materials

Activated carbons were texturally and chemically characterized by the experimental techniques listed below (Table 1) (Stoeckli and Centeno, 1997; Moreno and Giraldo, 2000; Silvestre-Albero et al., 2001; Thommes and Cychosz, 2014; Thommes et al., 2015; Alves et al., 2016).

**Table 1:** Techniques used in the characterization of activated carbons.

**Experimental technique**	**Conditions**	**Obtained parameters**
Adsorption isotherms of N_2_ at 77 K	Sample: 100 mg	Surface área (S_BET_)
		Micropore volume Vo (N_2_)
	Vacuum degassing: 423 K, 24 h.	
	Autorsorb IQ2 (Quantachrome Instruments)	
Boehm titration	Sample: 100 mg	- Oxygenated surface groups content: Carboxylic, Lactonic and Phenolic - Total acidity - Total basicity
	Solutions: NaOH, Na_2_CO_3_, NaHCO_3_ NaOH and HCl 0.1 M	
	Volume solutions: 25 mL	
	Temperature: 298 K	
	Contact time:48 h	
	N_2_ was bubbled over the solutions	
Point zero charge-pH _PZC_	Noh and Schwarz mass Titration Method (Noh and Schwarz, 1990)	pH _PZC_
	Samples: Between 0.050 and 0.300 g	
	Solution: 10 mL NaCl 0.1N	
	Temperature: 298 K	
	Time: 48 h	
Elemental analysis	CHNS elemental microanalyzer, with LECO Micro TruSpec detection system	Element content:- Carbon content - Nitrogen content - Hydrogen content - Oxygen content
Infrared spectroscopy (DR FTIR)	Sample: 100 mg	Chemical groups presents in materials
	Mixed with KBr	
	Diffuse reflectance cell	
	Thermo-Nicolet 6,700 FT-IR	
Immersion calorimetry	Sample: 100 mg	Immersion enthalpy
	Temperature: 298 K	
	Volume= 8 mL	
	Immersion liquids: C_6_H_6_ and H_2_O	
	Equilibrium time of base line: 1 h	
	Electrical calibration	
	Calvet type heat conduction calorimeter	

### CO_2_ Adsorption at 273 K and 1 Bar

To determine the adsorption isotherms of CO_2_ at 273 K and 1 Bar, a commercial semi-automatic sortometer Autosorb IQ2 (Quantachrome Instruments) was used, simultaneously a calorimeter coupled to the sortometer was used to measure the energy changes involved in each point of the isotherm, 100 mg of activated carbon were used, the samples were degassed at 423 K for 24 h, until the system reached a pressure between 10^−5^ and 10^−6^ Bar Simultaneously, the calorimetric signal was allowed to stabilize and injections of the adsorbate were carried out, waiting for the time necessary to reach the equilibrium between the system components, so the volumes of gas adsorbed and the heat involved in each injection were simultaneously recorded.

## Results and Discussion

### Textural Characteristics

**Figure 1:**
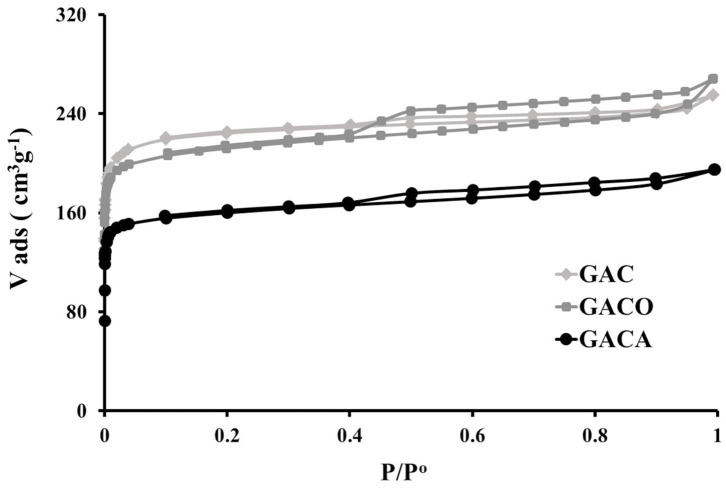
N_2_ adsorption isotherms at 77K for prepared samples.

The nitrogen adsorption isotherms are presented in Figure 1, it can be seen that the experimental impregnation and carbonization conditions used allowed obtaining micro-mesoporous solids, represented by type IV isotherms with H4 hysteresis loops, which are characterized by not presenting a steep slope in the adsorption branch at high pressures, which generates a small loop and almost horizontal adsorption-desorption branches, the H4 loop is the adsorption branch resulting of a combination of I and II isotherms types, showing a pronounced uptake at low p/p^0^ being associated with the filling of micropores. H4 loops are often found in micro-mesoporous carbons, according to the IUPAC Technical Report classification in 2015 and other authors (Thommes and Cychosz, 2014; Thommes et al., 2015).

The apparent surface areas were calculated from the BET equation, the micropore volume Vo (N_2_) and the narrow microporosity volume Vn (CO_2_) (Pores <0.7 nm), were obtained by applying the Dubinin equation -Radushkevich to nitrogen adsorption data (Liquid N_2_ density = 0.808 g cm^−3^) and carbon dioxide adsorption data (Liquid CO_2_ density = 1.023 g/cm^−3^), respectively. The total pore volume V_t_ was calculated from the adsorbed volume at a relative pressure of 0.99, and the mesopore volume by difference. Usually, in the case of activated carbons, the linearity interval of the representation of the BET equation is limited to the relative pressures between 0.05 and 0.35 [25-26], but with the aim of reducing any subjectivity in evaluating monolayer capacity the procedure proposed by Rouquerol et al. (2007) and ratified by IUPAC Technical Report in 2015 was used to determine the range of relative pressures, this is based on the following criteria: (1) the quantity C should be positive (i.e., a negative intercept on the ordinate of the BET plot is the first indication that it is not the appropriate range); (2) application of the BET equation should be restricted to the range where the term n(1–p/p^0^) continuously increases with p/p^0^; (3) the p/p^0^ value corresponding to n_m_ should be within the selected BET range.

Table 2 shows the textural parameters of activated carbons, it is observed that the BET area for porous solids is between 634 and 875 m^2^g^−1^ and the micropore volume is between 0.25 and 0.34 cm^3^g^−1^. The data of the textural parameters are comparable with those reported for activated carbons obtained from lignocellulosic residues with areas between 150 and 2,700 m^2^g^−1^ and pore volumes between 0.042 and 1.6 cm^3^g^−1^ (Jagtoyen et al., 1993; Molina-Sabio et al., 1996; Nakagawa et al., 2007; Juárez-Galán et al., 2009; Zuo et al., 2009). In Figure 1 and Table 2, the incidence of chemical treatments performed on the textural characteristics of the adsorbent material can be observed, it is evident that there was a decrease in the apparent surface area and the pore volume in the chemically modified carbon, this behavior can be explained taking into account that oxidation with HNO_3_ generates the reaction of this agent with the carbon atoms that there are in the openings of the pores or on the edges of the graphene layers of the carbonaceous material, giving rise to the formation of surface oxygenated groups that are located at the edges of the pore openings (Noh and Schwarz, 1990; Figueiredo et al., 1999; Figueiredo and Pereira, 2010), these mentioned facts generate a blockage in the porous structure of the material and it limits the access of the nitrogen molecule to the porous network, which generates a decrease in the BET area by 9.0 % and the pore volume by 5.9%, as seen in this work. Additionally, treatment with HNO_3_ can lead to a collapse in the carbonaceous structure, which results in a widening of the porosity, and therefore in an increase in the volume of mesopores (Noh and Schwarz, 1990; Figueiredo et al., 1999; Figueiredo and Pereira, 2010) as it can be evidenced in Table 2 in the GACO sample.

**Table 2:** Textural parameters for carbonaceous materials obtained from the N_2_ adsorption isotherms at 77 K.

**Nitrogen adsorption at 77 K**				
**Sample**	**S_**BET**_ (m^**2**^g^**−1**^)**	**V_**O**_ (cm^**3**^g^**−1**^)**	**V_**meso**_ (cm^**3**^g^**−1**^)**	**V_**0.99**_ (cm^**3**^g^**−1**^)**
GAC	865	0.34	0.06	0.40
GACO	787	0.32	0.10	0.42
GACA	634	0.25	0.07	0.32

Concerning the modification of the granular activated carbon with NH_4_OH, similarly, a smaller BET area and pore volume in the GACA material were evidenced, in relation to the non-chemically modified carbonaceous material (GAC). This fact is attributed to the obstruction caused by the new surface groups generated in the carbonaceous structure. Similar researching has shown that the reaction of carbonaceous materials with NH_4_OH develops groups such as amines, amides, nitriles, among others, at the edges of the graphene layers which block existing pores, reducing the surface area and pore volume (Plaza et al., 2007; Shafeeyan et al., 2011). In this work this decrease was of 26.7% and 26.5%, respectively. The results obtained also allowed establishing that the modification of the activated carbon (GAC) with NH_4_OH causes a greater effect on the textural parameters of the material, in comparison to the use of HNO_3_ in the process.

### Chemical Characteristics

Table 3 shows the results of the elemental analysis performed for the carbonaceous materials. It is important to clarify that the inorganic content of the solid, was included in the oxygen percentage, due to it was not performed an additional analysis of the composition of the samples. A decrease in carbon and hydrogen content is observed in the modified samples in regards to the non-chemically treated material, which is directly related to the chemical attack produced by the agents used for the modification in the carbonaceous structure; in this sense, different authors have previously reported that the treatment of activated carbon with HNO_3_, generates a decrease in the content of fixed carbon and hydrogen in the material as a result of the increase in volatile matter, due to the fact that it can generate the formation of humic substances (Figueiredo et al., 1999; Plaza et al., 2007; Zuo et al., 2009; Figueiredo and Pereira, 2010; Shafeeyan et al., 2011), on the other hand, the treatment of activated carbons with NH_4_OH, gives rise to the formation of volatile substances, for example in the reaction with ammonia, ether like oxygen surface groups are easily replaced by –NH– on the carbon surface that through dehydrogenation reaction could readily lead to imine and pyridine functionalities, this fact can to explain the decrease of carbon and hydrogen content in the materials (Saleh et al., 2010; Heidari et al., 2014). It is also observed that the reaction of the GAC sample with HNO_3_ generated an increase in the oxygen content of the material due to the oxidation of the surface, and the appearance of nitrogen, which could be added to the surface of the carbon through a similar reaction to the one of the benzene nitration, in which the mechanism involves the formation of the highly reactive ion, nitronium (${\text{NO}}_{2}^{-}$), which can form a nitrated product that is attached to the carbonaceous structure (Figueiredo et al., 1999; Zuo et al., 2009; Figueiredo and Pereira, 2010). In the case of treatment with NH_4_OH, it is important to highlight that this not only generates a higher nitrogen content on the surface of activated carbon, but it also increases the oxygen content, which has a direct effect on the CO_2_ adsorption capacity of the material, as it will be seen later.

**Table 3:** Results of the elemental analysis of carbonaceous materials.

**Elemental analysis**				
**Sample**	**%C**	**%H**	**%N**	**%O**
GAC	81.4	2.1	–	16.5
GACO	66.1	1.3	1.2	31.4
GACA	63.6	1.2	2.4	32.8

**Figure 2:**
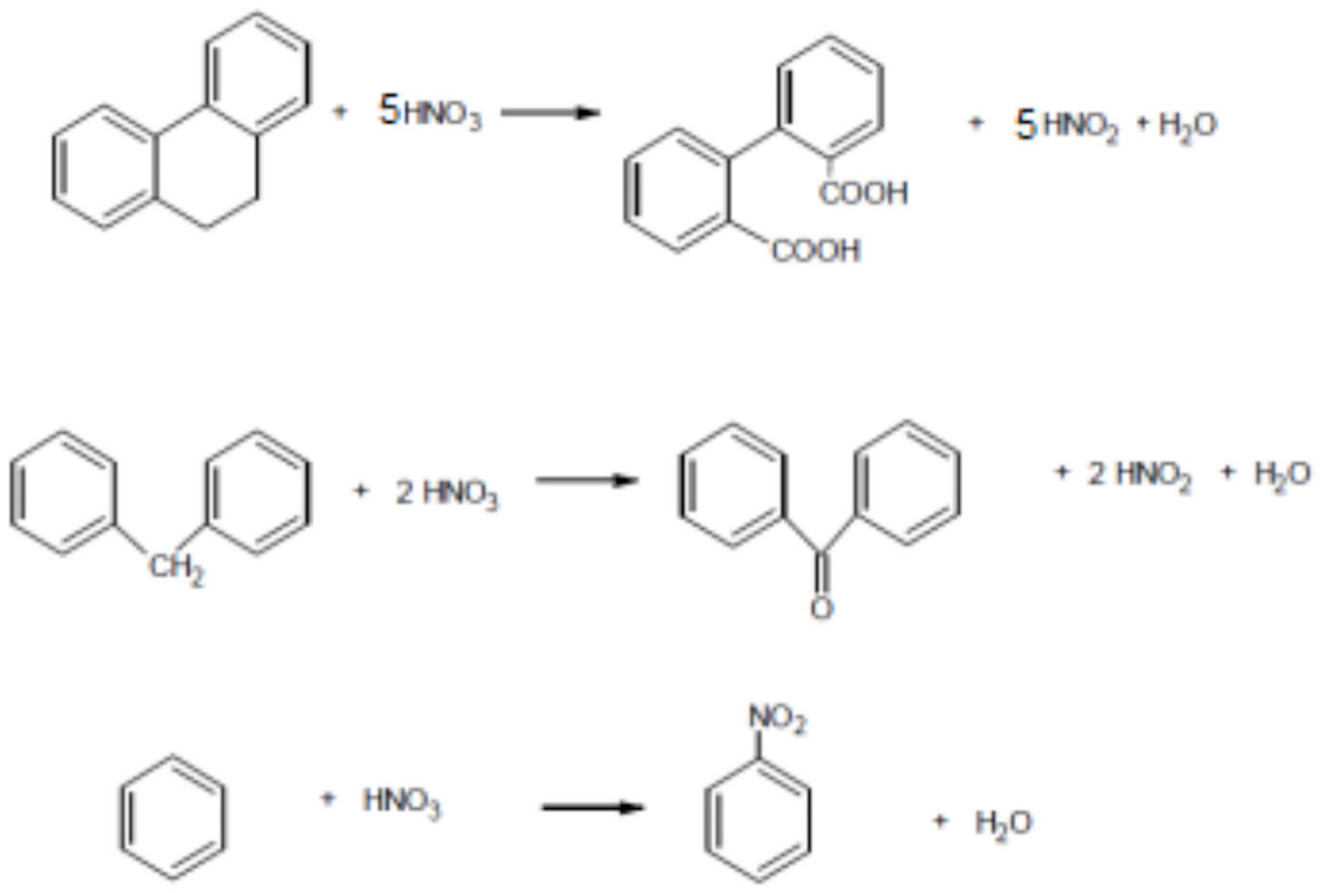
Effect of HNO_3_ treatment on the surface of an activated carbon (Chingombe et al., 2005).

Table 4 presents the surface groups content of carbonaceous materials, total acidity, total basicity and pHpzc. In order to determine the quantity and types of oxygenated groups located on the carbonaceous materials surface samples were immersed in NaOH, HCl, Na_2_CO_3_, and NaHCO_3_ 0.1M solutions. The most commonly used bases are NaHCO_3_ (pKa = 6.37), Na_2_CO_3_ (pKa = 10.25), and NaOH (pKa = 15.74). According to Boehm, the carboxylic groups are only titrated by NaHCO_3_, the difference between the acidity valued by NaHCO_3_ and Na_2_CO_3_ corresponds to the lactone content, and the phenolic groups and carbonyl groups are obtained from the difference between the acidity registered with NaOH and Na_2_CO_3_. Finally, hydrochloric acid gives an estimate of the total basicity of the material and NaOH gives an estimate of the total acidity of the material (Boehm, 2002). It is observed that the GACO sample has a higher content of carboxylic, lactonic, and phenolic groups with respect to the starting material, due to this the nature of the surface is acidic with a pHpzc of 6.2, it is evident that the oxidation treatment with HNO_3_ genearates the formation of acidic functional groups on the surface of activated carbon, this fact was explained previously and agrees with the results obtained in the elemental analysis carried out on the material. Some of the mechanisms by which oxygenated surface groups are formed as a result of nitric acid treatment have been reported by Chingombe et al. (2005) (Figure 2).

**Table 4:** Oxygenated surface groups content determined by the Boehm method, and the pH at the point of zero charge for the carbonaceous materials.

**Sample**	**Carboxylic groups mmolg^**−1**^**	**Lactonic groups mmolg^**−1**^**	**Phenolic groups mmolg^**−1**^**	**Total acidity mmolg^**−1**^**	**Total basicity mmolg^**−1**^**	**pHpzc**
GAC	0.08	0.10	0.16	0.34	0.07	7.8
GACO	0.12	0.21	0.35	0.68	0.18	6.2
GACA	0.05	0.05	0.11	0.21	0.28	8.4

From the results it is possible to establish that the modification of the activated carbon with HNO_3_ doubled the acidity of the original material, a fact that is directly related to the appearance of oxygenated acidic superficial groups such as carboxylic acids (Noh and Schwarz, 1990; Figueiredo et al., 1999; Zuo et al., 2009; Figueiredo and Pereira, 2010; Saha and Kienbaum, 2019). In the treatment of activated carbon with ammonium hydroxide, it is observed that the content of carboxylic, lactonic and phenolic groups was reduced, due to the reaction of these with ammonia to produce nitrogenous compounds and increase the basic character of the surface of activated carbon as it is evidenced in the increase in pH_PZC_ to 8.4 in the GACA sample. Moreover, it is possible to observe that the treatment with NH_4_OH generated a decrease in the acidity of the porous solid GAC and a four-fold increase in its basicity, which can be related to the incorporation of nitrogen in the carbonaceous structure and in general of electron-donating groups, together with π electrons delocalized, which increase the electron density of the graphene layers (Saha and Kienbaum, 2019).

**Figure 3:**
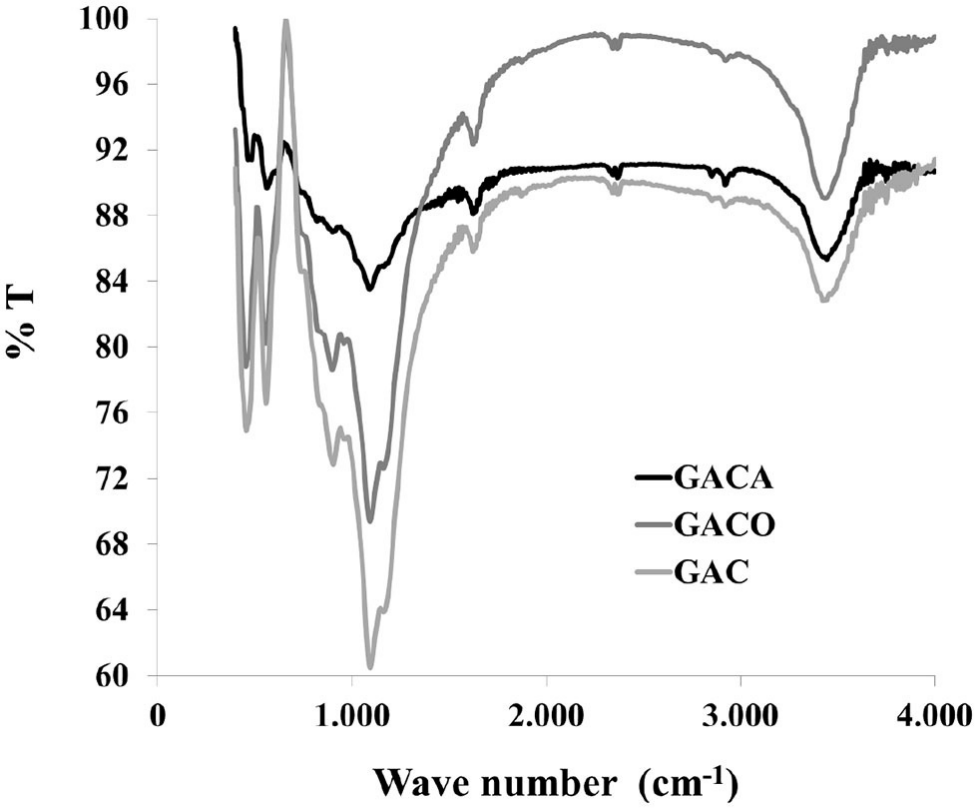
FTIR spectra for carbonaceous materials.

Figure 3 shows FTIR spectra of carbonaceous materials where it is possible to distinguish three bands of interest: in all samples are observed out-of-plane aromatic C–H vibrations (890, 820, 760 cm^−1^), likewise a band of different intensity located between 900 and 1,500 cm^−1^, as it has been discussed in similar studies (Meldrum and Rochester, 1990; Dandekar et al., 1998) in this region it is difficult to assign bands with certainty since there is overlap of the C-O stretch of different surface groups, in this sense, assignments have been made to C-O vibrations in esters (1,100–1,250cm^−1^), carboxylic acids and cyclic anhydrides (1,180–1,300 cm^−1^), lactones (1,160–1,370 cm^−1^), ethers (942–1,300 cm^−1^), cyclic ethers (1,140 cm^−1^), phenolic groups (1,180–1,220 cm^−1^), and epoxides (1,220 cm^−1^) (Meldrum and Rochester, 1990; Dandekar et al., 1998), with the oxidation process in the GACO sample is observed a slight increase in the intensity and width of this band in comparison to the CAG sample, which can be associated to the increase of oxygenated groups on the carbon surface. With regard to GACA sample is observed that the intensity of the peak decreases in comparison with CAG sample and this behavior is an evidence of the consumption of the oxygenated groups in the reaction with NH_4_OH, ratifying the results obtained by Boehm titrations. Likewise there is a peak around 1,600 cm^−1^, which is characteristic of carbonaceous materials, it can be attributed to polyaromatic C= C vibration in carbons with sp^2^ hybridization, other relevant vibrations can observed to this wavelength include carboxyl -carbonates (1,590 cm^−1^), quinones and hydroxyquinones (1,550–1,675 cm^−1^), and asymmetric stretches of carboxylate anions between 1,525 and 1,623 cm^−1^, the third peak located between 3,100 and 3,700 cm^−1^ is characteristic of the vibration of the -OH stretch of hydroxyl, carboxylic and phenolic groups (Meldrum and Rochester, 1990; Dandekar et al., 1998), in this region a significant increase in the intensity of the peak is observed with the oxidation of the activated carbon (sample GACO) and a decrease in the signal after the reaction of the solid with NH_4_OH (sample GACA), which shows the modification of the surface chemistry of the starting solid (GAC sample) with the treatments that it was subjected.

### Immersion Calorimetry

Immersion calorimetry is an experimental technique that allows a quantification of the energy change associated to the interaction of a solid with a liquid in which it is submerged and in which the solid is insoluble and they do not react at a constant temperature and pressure. This technique is very useful in the characterization of adsorbent materials, due to the changes in the enthalpy of immersion are directly associated with variations in the surface area, the chemical surface and the microporosity of the porous materials, in this sense, its versatility has been evidenced in several studies, when wetting liquids with different chemical characteristics are used (Stoeckli and Centeno, 1997; Moreno and Giraldo, 2000; Silvestre-Albero et al., 2001).

**Figure 4:**
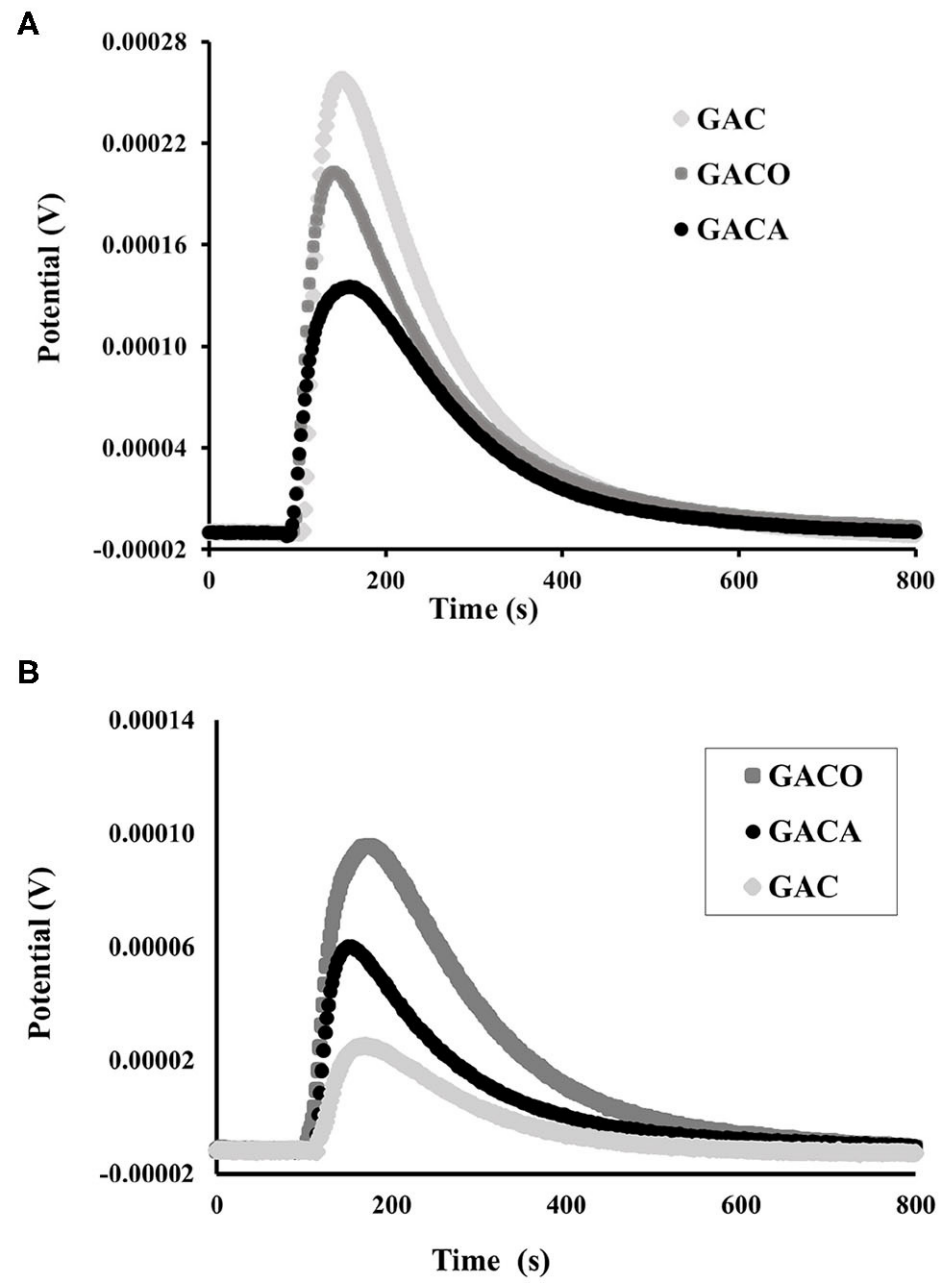
**(A)** thermogram of the immersion of samples in benzene, **(B)** thermogram of the immersion of samples in water.

In Figure 4A the thermogram of the immersion of the activated carbon in benzene is shown, this non-polar molecule does not chemically interact with the solid, the molecules of this liquid enter the carbonaceous structure, accessing the porosity and forming a layer on the solid and therefore the energy associated with the interaction process is directly related to the available surface area in porous materials (Acevedo et al., 2015). On the other hand, in Figure 4B, it can be observed that water due to it polar nature interacts mainly with the oxygenated surface groups located at the polar sites at the edges of the graphene layers, because it allows to evaluate the polarity and hydrophobicity of the surface of a solid. Consequently, the magnitude of the immersion peaks observed in Figures 4A,B is directly related to the values of the enthalpies obtained and therefore with the surface areas, the polarity and hydrophobicity of the activated carbon prepared during the study. Thus, it can be seen that the GAC material has the highest immersion peak in benzene (Figure 4A) and, accordingly, it is the porous solid that has the largest BET surface area, as shown in Table 2, in the same way its relation is evidenced in the GACO and GACA materials.

Table 5 shows the enthalpies of immersion in benzene and water and the hydrophobic factor that is calculated as the ratio between the immersion enthalpy of samples in benzene and the immersion enthalpy in water. All the enthalpies of immersion in benzene and water are exothermic, in relation to the superficial process that takes place between the solid and the liquid.

**Table 5:** Immersion enthalpies in benzene and water for carbonaceous materials.

**Sample**	**–ΔH_**imm**_ C_**6**_H_**6**_ (Jg^**−1**^)**	**–ΔH_**imm**_ H_**2**_O (Jg^**−1**^)**	**Hydrophobic factor (–ΔH_**imm**_ C_**6**_H_**6**_/ -ΔH_**imm**_H_**2**_O)**
GAC	108	48	2.25
GACO	84	68	1.23
GACA	75	56	1.34

The enthalpies in benzene, for the set of solids obtained, are between −75 and −108 J g^−1^ and in water they are between −48 and −68 J g^−1^. The results show that the enthalpies of immersion in benzene correlate with the BET surface areas (Table 2) of the activated carbons, obtaining a higher enthalpy for the GAC material whose area is the largest of the three solids and the lowest enthalpy value for the GACA sample whose area is the smallest, which is consistent since the greater the surface area is there is greater access to the benzene molecule. In regards to the hydrophobic factor, it is observed that the treatments to which the GAC activated carbon was subjected generated a decrease in the hydrophobicity of the material, due to the fact that its chemical surface was enriched by oxygenated and nitrogenous groups which interact with the molecules of water increasing the affinity of activated carbon with water. In this sense, it is determined that the magnitude of the immersion enthalpy of the three samples in water, correlate with the acidity of the material, as a result, an increase in this parameter with the acidity of the solids is found, as it has been shown in other studies (Stoeckli and Centeno, 1997; Silvestre-Albero et al., 2001; Acevedo et al., 2015).

### CO_2_ Adsorption

**Figure 5:**
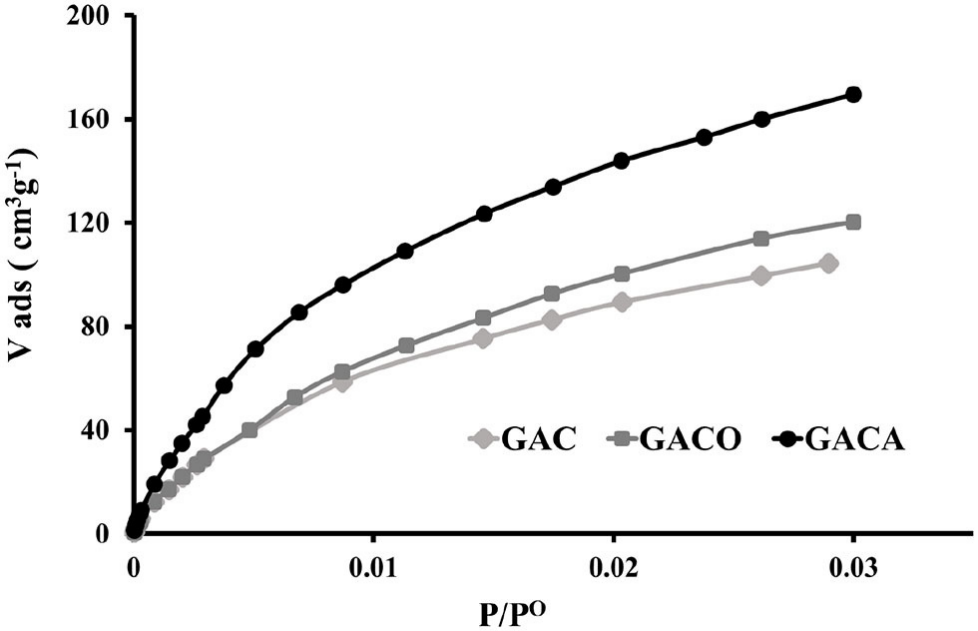
CO_2_ adsorption isotherms at 273K and 1 Bar for prepared samples.

Figure 5 shows the CO_2_ adsorption isotherms at 273 K and 1 Bar that were obtained for carbonaceous materials, the isotherms obtained are Type I according to the IUPAC classification. It can be seen that the treatments with HNO_3_ and NH_4_OH generated an increase in the CO_2_ adsorption capacity of the carbon, which can be associated with the chemical enrichment of the surface with surface groups that favor the affinity of the solid and the adsorbate.

The energy study of the adsorption processes is important to establish the magnitude of the interaction between adsorbate and adsorbent. In order to obtain information about CO_2_ adsorption in the prepared activated carbon, a direct measurement of the heat of adsorption generated at each point of the gas isotherm at 273K and 1 Bar was performed using the adsorption calorimetry, these measurements provide information about the energetic heterogeneity of an adsorbent in the adsorption.

**Figure 6:**
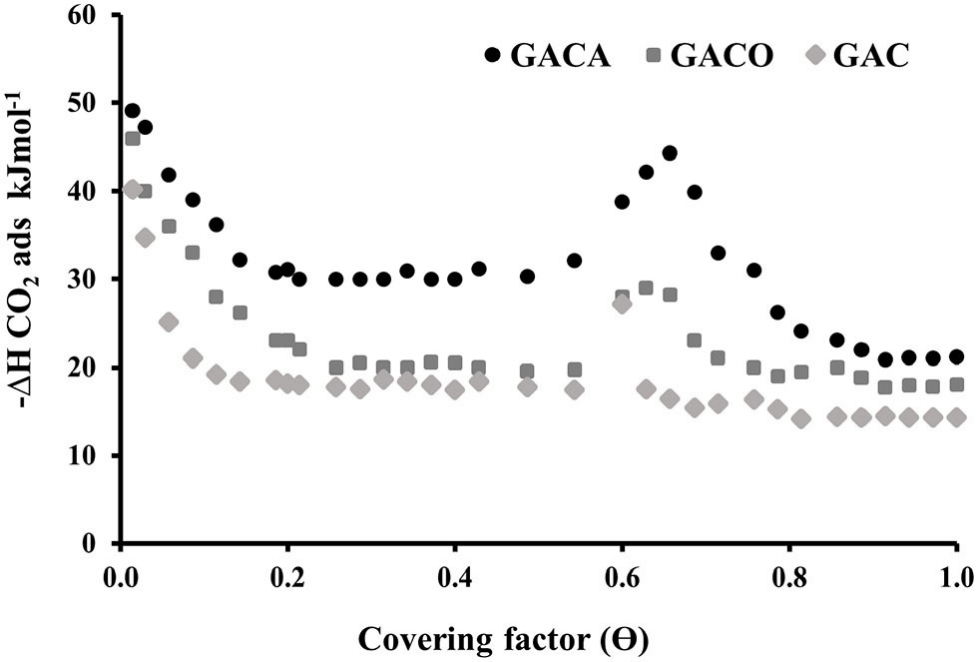
Differential heat of adsorption of CO_2_ for the prepared samples.

Figure 6 shows the differential heat of adsorption regarding the adsorbed amount of gas and the coverage, it is observed that the samples follow the next increasing order of heat of adsorption in the entire coverage range (O) GAC < GACO < GACA, which is directly related to the CO_2_ adsorption capacities of these materials, taking into account that the adsorption of gas on samples increased in the same order. For all of the three samples it is evident that at an adsorbed amount or coverage close to zero, the heat of adsorption has the highest values of the entire range, this heat is associated with the presence of strong interactions between the CO_2_ molecule and the narrow micropore walls, it is also related to the fact that the highest energy sites are filled at low coverage. Additionally, in the case of the GACO and GACA samples, the magnitude of the interaction increases due to the incorporation of oxygenated and nitrogenous groups on the carbonaceous surface which, at the same time, favors the affinity of the solid, in addition it increases molecular interactions between the adsorbed CO_2_, which means a greater heat of adsorption than that coverage (Maia et al., 2018). Subsequently it is observed that the adsorption heat significantly decreased to a coverage of 0.58 for the three samples, which evidences the occupation of adsorption sites in the materials, then between a coverage of 0.58 to 0.75 a is observed a maximum peak in the heat of adsorption, this behavior has been associated with adsorbate-adsorbate interactions that are responsible for the energy maxima at quantities between 0.60 and 0.80 (Rouquerol et al., 1999). From the above-mentioned coverings, the heats of adsorption decrease to almost constant levels, which is probably due to the occupation of low potential adsorption sites that were previously occupied at O> 0.85. In general terms, it is evident that the magnitude of the initial adsorption enthalpy in the three materials is closely related to the high-energy filling of pores of the microporous region present in the solids, followed by an energy drop corresponding to the filling of the larger pores. Additionally, it is important to highlight that the obtained enthalpy values do not exceed 50 kJmol^−1^, which indicates that a process of physical adsorption of CO_2_ is carried out in solids.

**Figure 7:**
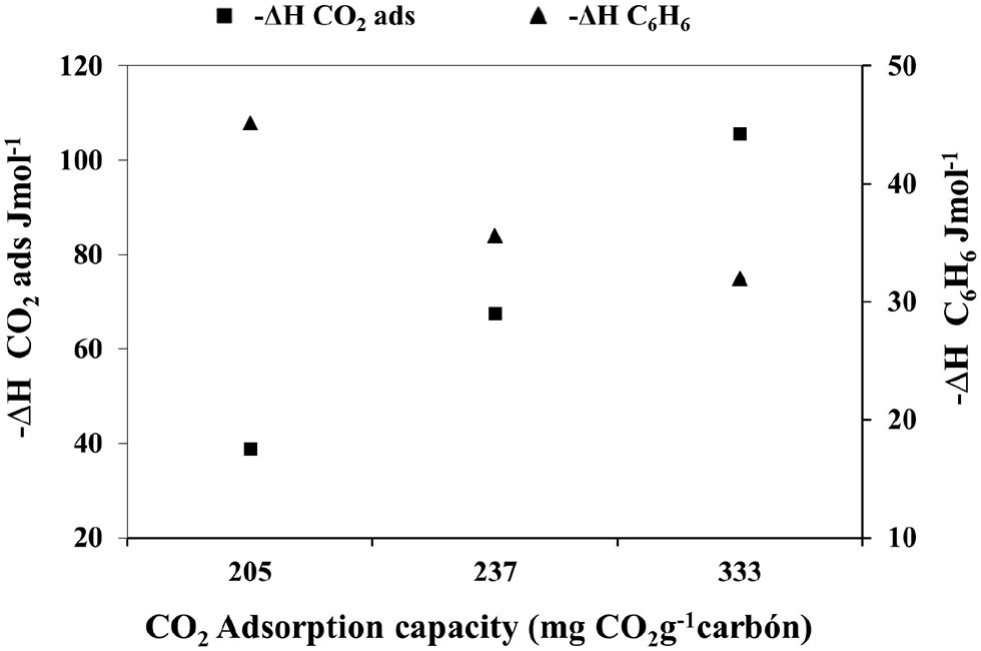
Relationship between the CO_2_ adsorption capacity of activated carbon, enthalpy of adsorption of CO_2_ and enthalpy of immersion in benzene.

Figure 7 shows the relationship between the CO_2_ adsorption capacity in regards to the enthalpy of adsorption of CO_2_ and the enthalpy of immersion of the carbonaceous materials in benzene. It is observed that at a higher CO_2_ adsorption capacity there is an increase in the enthalpy value of this gas, which is consistent since there is a greater interaction between the adsorbate and the adsorbent. Besides, it is possible to appreciate the increase in the CO_2_ adsorption capacity of the activated carbon as the immersion enthalpy in benzene decreases, in order to analyze this behavior, it is important to highlight that immersion in benzene enthalpy is directly related to the accessible surface area of activated carbon, which means that, the greater the enthalpy value, the greater the surface area of the materials; in this sense, Figure 7 illustrates that the removal of the gas does not depend on the accessible area of the carbonaceous materials, instead, it is related to the chemical nature of the surface and to energy aspects of interaction.

**Figure 8:**
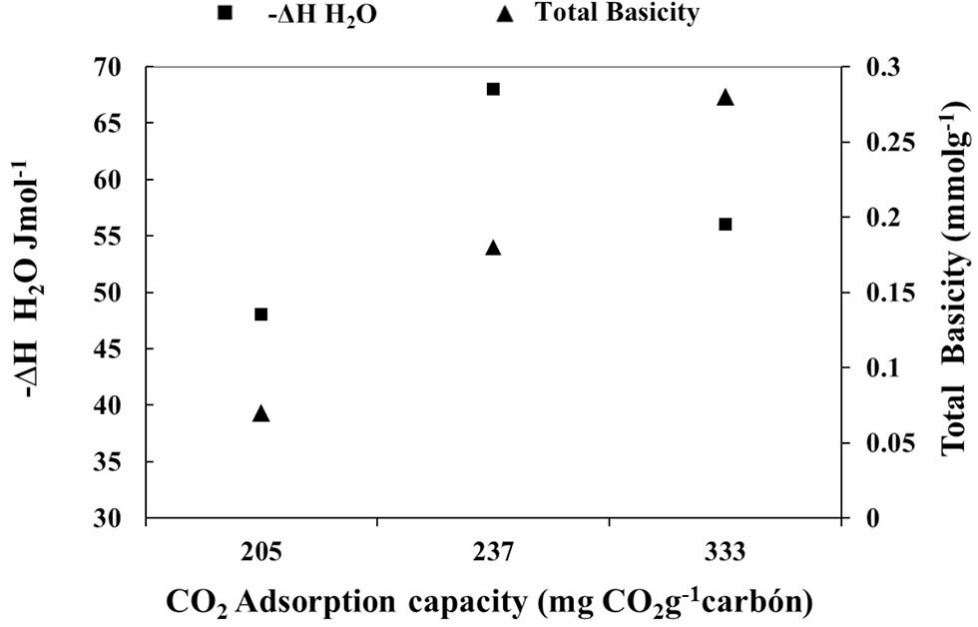
Relationship between the CO_2_ adsorption capacity of activated carbon with the enthalpy of immersion in H_2_O and the total basicity of solids.

In order to establish which characteristics of the porous solids determine the adsorption of CO_2_, Figure 8 was plotted in order to show the relationship between the CO_2_ adsorption capacity of activated carbons, the enthalpy of immersion in H_2_O and the total basicity of solids. The tendency to increase the CO_2_ adsorption capacity of the materials is evidenced as the total basicity of these increases, which is consistent with the chemical nature of the gas, since it behaves as a Lewis acid. Concerning the enthalpy in water and CO_2_ adsorption, an initial increase in calorimetric data and then a decrease is shown, this can be associated with the fact that the quantification of the total basicity of an activated carbon is complex, taking into account that not only the functional groups determine the mentioned parameter but also the delocalized electrons present in the graphene layers influence the process (Stoeckli and Centeno, 1997; Figueiredo et al., 1999) therefore, the enthalpy of immersion in water does not keep a clear correlation with the basicity.

Table 6 shows the CO_2_ adsorption capacities of activated carbons prepared in this work, the range of this parameter is from 205 to 333 mg g^−1^, these data are satisfactory, considering the fact that in other studies the adsorbed amounts have been between 43 and 400 mg g^−1^ in adsorbent materials such as: zeolites, carbon fibers, MOF and activated carbon (Plaza et al., 2010, Carruthers et al., 2012; Cho et al., 2012; Sevilla and Fuertes, 2012; Wahby et al., 2012; Yang et al., 2012; An et al., 2013; Liu et al., 2013; Jang et al., 2018; Querejeta et al., 2018). Taking into account the CO_2_ adsorption capacities of the prepared activated carbon, it can be affirmed that the modifications made with HNO_3_ and NH_4_OH were effective since they generated an increase of up to 61.56% in the adsorbed amount of this gas on the carbonaceous materials, This increase is associated with the incorporation of nitrogen groups that act as electron donors and carboxylic groups that are capable of establishing Lewis acid-base interactions with the CO_2_ molecule, because these groups not only have a carbonyl group that can act as a Lewis base toward the carbon atom (Lewis acid) of the molecule, but also has an acidic proton that can act as a Lewis acid toward the oxygen atom (Lewis bases) of the CO_2_ molecule (Bell et al., 2003).

**Table 6:** CO_2_ adsorption capacity (mgCO_2_g^−1^) at 273K and 1 bar for the samples.

**Sample**	**GAC**	**GACO**	**GACA**
**mg CO**_**2**_ **g**^**−1**^**carbon**	205	237	333

## Conclusions

The methodology used to prepare activated carbons allowed obtaining micro-mesoporous carbonaceous materials, with BET surface areas between 634 and 865 m^2^g^−1^ and pore volumes between 0.25 and 0.34 cm^3^g^−1^. It was evidenced that the solids presented textural and chemical characteristics useful for the removal of CO_2_ at 273 K and 1 Bar, achieving an adsorption capacity of this gas between 205 and 333 mgCO_2_ g^−1^carbon, which was satisfactory when it was compared with other porous solids prepared in other investigations.

The experimental results showed that the adsorption of CO_2_ in the carbonaceous materials was not related to the textural characteristics of the solids such as the BET surface area and the pore volume but depended on the chemical surface of the activated carbons, since this determined the interaction of the surface with the gas molecule. In this sense, by increasing the oxygenated and nitrogenous groups on the surface of the activated carbons, it was possible to increase the affinity of the solid for the CO_2_ molecule which had acidic characteristics, and therefore the electron-donating groups favored its adsorption.

The energy characterization of the CO_2_ adsorption process, allowed establishing that the enthalpy values associated with the process of the surface covering of the solid by the gas did not exceed 50 kJmol^−1^, which reflected that the adsorption that was carried out in the carbonaceous materials studied before was of a physical nature. These results were important since they showed that although carbon was modified to enrich the superficial chemical with groups that increased the interaction of the surface of the solids with the CO_2_ molecule, these interactions were not a strong feature of the order of the chemical bond, which would facilitate their subsequent regeneration.

## Data Availability Statement

The datasets generated for this study are available on request to the corresponding author.

## Author Contributions

All authors listed have made a substantial, direct and intellectual contribution to the work, and approved it for publication.

## Conflict of Interest

The authors declare that the research was conducted in the absence of any commercial or financial relationships that could be construed as a potential conflict of interest.
